# Determinants of Age‐Appropriate Childhood Immunization Uptake: A Cross‐Sectional Study of Caregiver, Child, Knowledge, Access, and Attitudinal Factors

**DOI:** 10.1002/puh2.70421

**Published:** 2026-10-14

**Authors:** Aruwa Simon Ayigu, Stephen Olaide Aremu, Adamu Ishaku Akyala, Abdillahi Abdi Barkhadle

**Affiliations:** ^1^ Global Health and Infectious Diseases Control Institute Nasarawa State University Keffi Nigeria; ^2^ Rift Valley University Jimma Ethiopia

**Keywords:** caregiver education, child health, childhood immunization, health service access, vaccine uptake

## Abstract

**Background:**

Childhood immunization is among the most cost‐effective public health strategies for reducing under‐five morbidity and mortality. However, full immunization coverage for age remains suboptimal in many low‐ and middle‐income countries due to factors related to caregivers, children, health systems, and sociocultural contexts. The study aimed to assess the prevalence of full immunization for age and identify its associated determinants across multiple domains.

**Methods:**

A community‐based cross‐sectional study was conducted among 364 caregiver–child pairs. Full immunization for age was the outcome variable. Data were collected using a structured interviewer‐administered questionnaire assessing sociodemographic characteristics, child factors, caregiver knowledge and perceptions, access to health services, and attitudes and practices. Chi‐square tests identified associations, and variables significant at *p* < 0.05 were entered into multivariable logistic regression models to determine independent predictors.

**Results:**

Overall, 261 children (71.7%) were fully immunized for age. Caregiver education was a strong predictor: children of caregivers with primary (adjusted odds ratio [AOR] = 3.91), secondary (AOR = 3.30), and tertiary education (AOR = 4.71) were significantly more likely to be fully immunized than those with no education. Rural residence increased the odds of full immunization (AOR = 3.23), as did household size ≥6 (AOR = 2.06). Older child age strongly predicted full immunization, particularly among children aged 12–17 months (AOR = 5.42) and 18–23 months (AOR = 6.83). Health facility delivery nearly quadrupled the likelihood of full immunization (AOR = 3.96). Reliance on non‐health worker information sources reduced uptake, whereas shorter travel time to facilities, regular community immunization sessions, intention for catch‐up vaccination (AOR = 8.74), and positive community advocacy significantly increased coverage.

**Conclusion:**

Full immunization for age is shaped by caregiver education, child age, health service accessibility, and caregiver attitudes. Interventions should prioritize caregiver education, strengthen health‐worker‐led communication, promote facility‐based deliveries, and expand regular community immunization services to improve coverage.

## Graphical abstract

Full immunization for age was achieved by 71.7% of children. Caregiver education, health facility delivery, rural residence, regular community immunization services, and positive caregiver attitudes significantly increased coverage, highlighting the need to strengthen primary health care services, health education, and equitable access to routine childhood immunization.

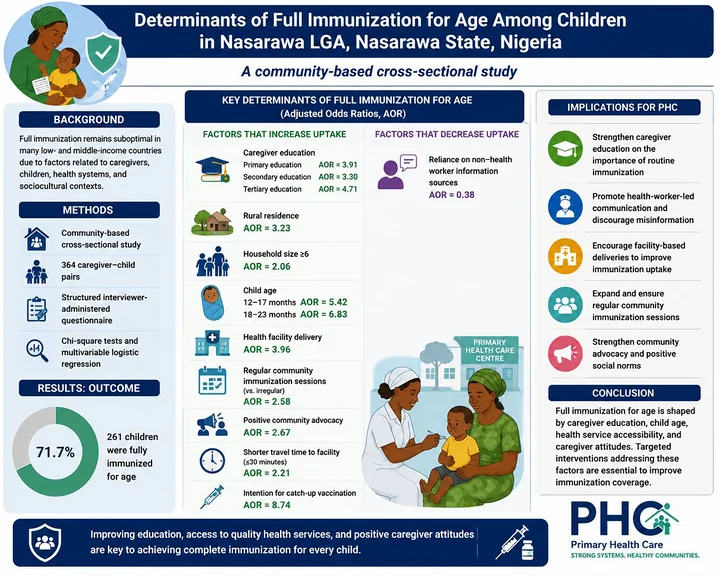

## 1 Introduction

Immunization is universally recognized as one of the most effective and economical public health interventions for preventing infectious diseases and reducing childhood morbidity and mortality [1, 2, 3, 4, 5]. Vaccines avert estimated millions of deaths globally each year and contribute significantly to improved child survival, cognitive development, and long‐term population health outcomes [2, 3]. Despite these benefits, many countries continue to experience suboptimal immunization coverage, particularly with respect to children being fully immunized for age according to national and World Health Organization (WHO) schedules [6, 7].

In low‐ and middle‐income countries, gaps in routine immunization persist due to a combination of demand‐side and supply‐side factors [7]. These include caregiver‐related characteristics such as education, knowledge, beliefs, and decision‐making autonomy; child‐related factors such as age and place of delivery; and health system factors, including accessibility, quality of services, and reliability of vaccine supply [8, 9, 10]. Sociocultural norms and community‐level influences further shape caregivers’ perceptions and practices regarding childhood vaccination [11, 12, 13, 14, 15].

Caregiver education has consistently emerged as a strong predictor of childhood immunization uptake [11, 16]. Educated caregivers are more likely to understand the purpose and schedule of vaccines, recognize the risks of vaccine‐preventable diseases, and navigate health services effectively [17]. Conversely, limited education may exacerbate misinformation, fear of adverse events, and reliance on informal information sources such as family members or religious leaders, which may undermine confidence in vaccines [16, 17, 18]. The role of information sources is, therefore, critical, as health workers are often the most trusted and accurate conveyors of immunization‐related information [19, 20].

Child‐specific factors also play a crucial role in immunization status. Age is particularly important, as younger children may not yet be eligible for all scheduled vaccines, whereas missed opportunities early in life can lead to incomplete immunization later [21, 22]. Place of delivery has been shown to influence early initiation and continuity of immunization, with health facility deliveries facilitating immediate linkage to routine immunization services, provision of vaccination cards, and caregiver counseling [23, 24].

Access to health services remains a major determinant of immunization uptake [10, 11, 12]. Physical distance to health facilities, transportation challenges, travel time, and the availability of regular outreach or community‐based immunization sessions all affect caregivers’ ability to complete vaccination schedules [16, 25]. Even where vaccines are provided free of charge, indirect costs such as transportation and opportunity costs related to time away from work may discourage caregivers from attending immunization sessions [9, 10, 11]. Health system factors, such as long waiting times, perceived disrespect from health workers, and vaccine stock‐outs, can further erode trust and reduce utilization of services.

Beyond knowledge and access, caregiver attitudes and practices significantly shape immunization behaviors [10]. Intentions to seek catch‐up immunization after missed doses, willingness to recommend immunization to others, and the influence of key decision‐makers within households and communities are powerful drivers of vaccine uptake. In many settings, immunization decisions are not made solely by mothers but are influenced by spouses, extended family members, community leaders, and religious authorities [12]. Understanding these dynamics is essential for designing culturally sensitive and community‐responsive interventions.

Given the multifaceted nature of immunization determinants, there is a need for comprehensive analyses that simultaneously examine sociodemographic, child‐related, knowledge‐based, access‐related, and attitudinal factors. This study therefore aimed to assess the prevalence of full immunization for age and identify its associated determinants across multiple domains. Evidence generated from this study is intended to inform targeted strategies to strengthen routine immunization programs, enhance caregiver engagement, and ultimately improve child health outcomes.

## 2 Methodology

### 2.1 Study Design and Setting

This study employed a community‐based cross‐sectional design to assess the immunization status of children and identify factors associated with full immunization for age. The study was conducted in 12 selected communities within Nasarawa local government area (LGA) of Nasarawa State, North‐Central Nigeria (Figure 1). Nasarawa LGA is administratively divided into 15 political wards, namely, Udenin Gida, Akum, Udenin, Loko, Tunga/Bakono, Guto/Aisa, Nasarawa North, Nasarawa East, Nasarawa Central, Nasarawa Main Town, Ara I, Ara II, Laminga, Kanah/Ondo/Apawu, and Odu.

**FIGURE 1:**
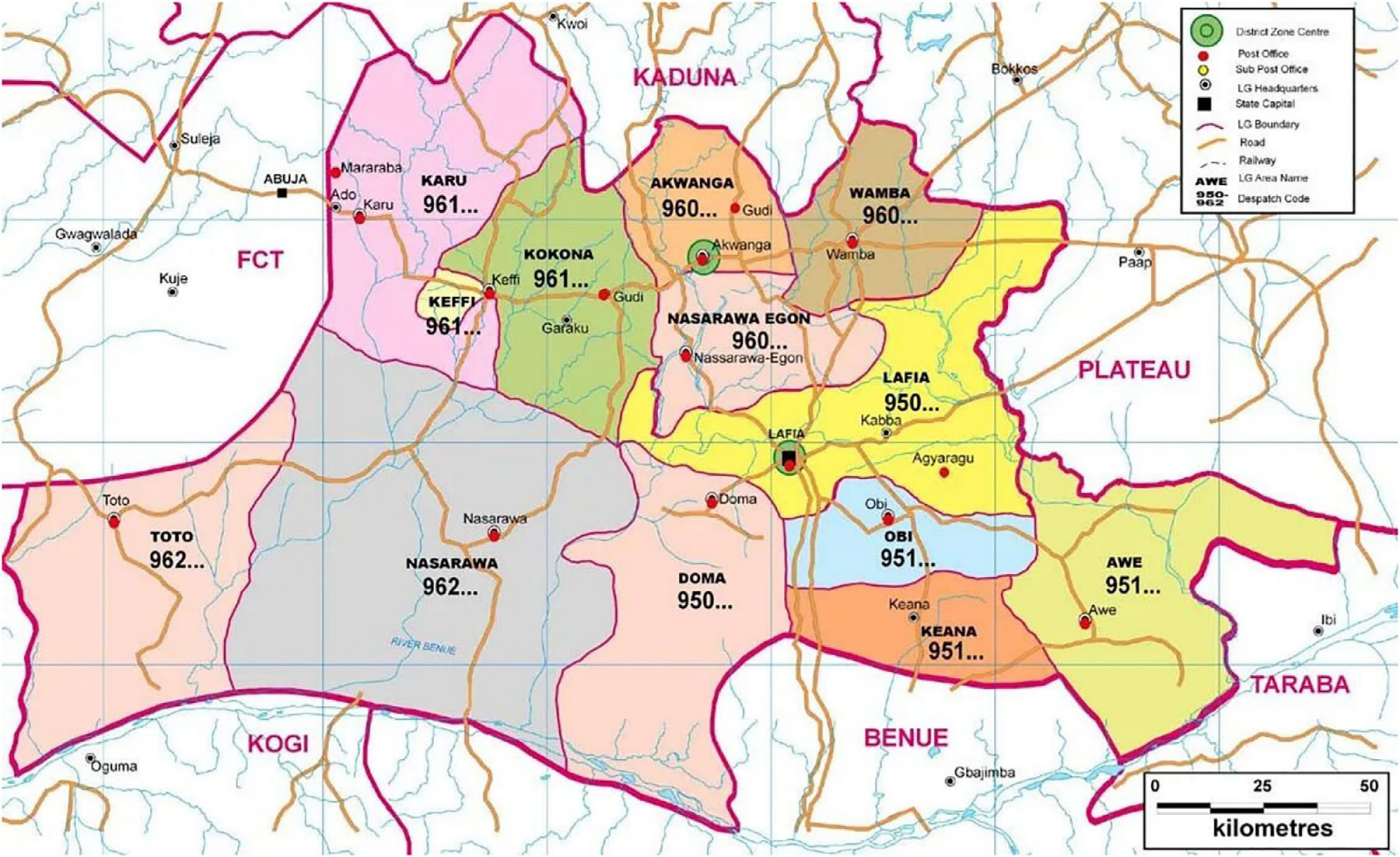
Image showing the study location (Nassarawa or Nasarawa State) used for the study in ash color, culled from Aremu et al. 2025 [25].

The study communities were selected using a multistage sampling technique. In the first stage, six wards were selected from the 15 wards using simple random sampling (balloting). In the second stage, two communities were randomly selected from each of the selected wards, yielding a total of 12 communities. Eligible households with children aged 12–23 months were subsequently identified using systematic household sampling. This multistage sampling approach ensured that communities from different geographical areas of the LGA were represented while minimizing selection bias.

Nasarawa LGA is served by a network of public and private health facilities that provide routine immunization services in accordance with Nigeria's National Expanded Programme on Immunization (EPI). These services target children from birth to 23 months of age and include vaccines against tuberculosis, poliomyelitis, diphtheria, pertussis, tetanus, hepatitis B, *Haemophilus influenzae* type b, pneumococcal disease, rotavirus, measles, yellow fever, meningitis A, and other nationally recommended antigens according to the Nigerian National Immunization Schedule.

Routine immunization services in the study area are delivered through fixed sessions at health facilities and periodic community outreach activities, particularly in hard‐to‐reach and underserved settlements. These outreach services are coordinated by the Nasarawa Local Government Primary Health Care Authority in collaboration with the Nasarawa State Primary Health Care Development Agency and the National Primary Health Care Development Agency to improve geographical access and equity in vaccine delivery. The community‐based design enabled the inclusion of both users and nonusers of routine immunization services, thereby providing a more representative assessment of childhood immunization coverage and its associated determinants within the LGA.

### 2.2 Study Population

The study population consisted of caregivers of children aged 0–23 months residing in the study area at the time of the survey. A caregiver was defined as any adult, most commonly the mother, who was primarily responsible for the child's day‐to‐day care and immunization decisions. Caregivers who had lived in the community for at least 6 months prior to the study were eligible to participate. Caregivers who were severely ill or unwilling to provide informed consent at the time of data collection were excluded.

### 2.3 Sample Size Determination and Sampling Technique

The sample size was determined using the Cochran single population proportion formula for estimating proportions in large populations:

$$n=\frac{Z^{2}\times p\times \left(1-p\right)}{d^{2}}$$

where n is the required minimum sample size. Z is the standard normal deviate corresponding to a 95% confidence level (1.96). p is the estimated prevalence of full immunization from previous similar studies. d is the margin of error (precision), set at 5% (0.05).

Assuming an estimated prevalence (p) of full immunization of 0.50, a conservative estimate commonly used when population prevalence varies across settings to maximize sample size, the initial sample size was calculated as follows:

$$n=\frac{{\left(1.96\right)}^{2}\times 0.50\times \left(1-0.50\right)}{{\left(0.05\right)}^{2}}$$

$$n=\frac{3.84\times 0.25}{0.0025}$$

n=384

To account for potential nonresponse or incomplete data, an adjustment of approximately 5% was applied:

$$\;n_{\mathrm{adjusted}}=384\times \left(1-0.05\right)\approx 364$$

Thus, a minimum sample size of 364 caregiver–child pairs was deemed sufficient to ensure adequate statistical power and precision for estimating the proportion of fully immunized children.

A multistage sampling technique was used to select participants. In the first stage, communities were selected using simple random sampling. In the second stage, households with eligible children aged 0–23 months were identified through household listing. Where more than one eligible child was present in a household, one child was selected by simple random sampling. The corresponding caregiver of the selected child was then interviewed.

### 2.4 Data Collection Instrument and Procedure

Data were collected using a structured interviewer‐administered questionnaire developed on the basis of relevant literature and national immunization guidelines. The questionnaire comprised five sections: sociodemographic characteristics of caregivers and households; child‐related factors; caregiver knowledge and perceptions regarding immunization; access to health services and perceived barriers; and caregiver attitudes and practices toward childhood immunization.

Immunization status was verified primarily through review of the child's immunization card. In cases where the card was unavailable, caregiver recall was used as a supplementary source of information. Data collection was conducted by trained research assistants who were fluent in the local language and familiar with the study context. The questionnaire was pretested in a community outside the study area, and necessary adjustments were made to improve clarity and consistency.

### 2.5 Study Variables and Operational Definitions

The dependent variable was child immunization status, categorized as *fully immunized for age* or *not fully immunized for age*. A child was considered fully immunized for age if they had received all vaccines recommended for their age according to the national EPI schedule at the time of the survey.

Independent variables included caregiver sociodemographic characteristics (age, education, marital status, occupation, household size, income, and place of residence), child factors (age, sex, birth order, and place of delivery), caregiver knowledge and perceptions, access to health services (distance, travel time, transport, and availability of immunization sessions), and caregiver attitudes and practices related to immunization.

### 2.6 Data Management and Statistical Analysis

Completed questionnaires were checked daily for completeness, accuracy, and consistency before data entry. Questionnaires with substantial missing or incomplete responses were excluded from the final analysis. Overall, 7% of the administered questionnaires were excluded because of incomplete data, resulting in a final analytical response rate of 93%. No imputation of missing values was performed, as only complete questionnaires were included in the final analysis. Data were coded and entered into IBM Statistical Package for the Social Sciences (SPSS) version 27.0 (IBM Corp., Armonk, NY, USA) for analysis. Descriptive statistics, including frequencies, percentages, means, and standard deviations, were used to summarize participants’ sociodemographic characteristics and immunization coverage. Bivariate analysis using the chi‐square test was performed to assess the association between independent variables and full immunization for age. Variables that were statistically significant at the bivariate level (*p* < 0.05) were included in a multivariable binary logistic regression model to identify independent predictors of full immunization for age while controlling for potential confounding factors. The results of the regression analysis were presented as adjusted odds ratios (AORs) with 95% confidence intervals (CIs). Statistical significance was established at *p* < 0.05.

## 3 Results

Table 1 shows that 261 (71.7%) children were fully immunized for age. Caregiver age, relationship to the child, marital status, occupation, and household income were not significantly associated with full immunization in bivariate analyses. However, caregiver educational level showed a strong and statistically significant association with immunization status (*χ* ^2^ = 12.13, *p* = 0.007). After adjustment, caregivers with primary, secondary, and tertiary education were significantly more likely to have fully immunized children compared with those with no formal education (AORs = 3.91, 3.30, and 4.71, respectively; all *p* < 0.001), demonstrating a clear educational gradient.

**TABLE 1:** Sociodemographic factors associated with child's immunization status.

Characteristics	Fully immunized for age		*χ* ^2^	*p* value	AOR (95% CI)	*p* value
	Yes (*n* = 261) Frequency (%)	No (*n* = 103) Frequency (%)				
**Age of caregiver (in years)**						
<20	4 (66.7)	2 (33.3)	7.428	0.059		
20–29	97 (64.2)	54 (53.8)				
30–39	121 (77.1)	36 (22.9)				
≥40	39 (78.0)	11 (22.0)				
**Relationship to child**						
Mother	225 (72.1)	87 (27.9)	6.277	0.099		
Father	24 (77.4)	7 (22.6)				
Grandparent	12 (63.2)	7 (36.8)				
Other (aunty)	0 (0.0)	2 (100)				
**Marital status**						
Married	230 (71.4)	92 (28.6)	0.104	0.747		
Not married	31 (73.8)	11 (26.2)				
**Educational level**						
None	35 (54.7)	29 (45.3)	12.129	0.007^*^	1	<0.001^*^
Primary	77 (77.8)	22 (22.2)			3.911 (1.893–8.080)	<0.001^*^
Secondary	106 (72.6)	40 (27.4)			3.298 (1.706–6.378)	<0.001^*^
Tertiary	43 (78.2)	12 (21.8)			4.705 (1.974–11.21)	<0.001^*^
**Occupation**						
Farmer	90 (76.9)	27 (23.1)	4.685	0.196		
Trading/Business	45 (64.3)	25 (35.7)				
Civil servant	29 (78.4)	8 (21.6)				
Housewife	97 (69.3)	43 (30.7)				
**Place of residence**						
Urban	39 (53.4)	34 (46.6)	15.036	<0.001^*^	1	<0.001^*^
Rural	222 (76.3)	69 (23.7)			3.231 (1.789–5.834)	
**Household size**						
2–3	81 (65.3)	43 (34.7)	8.809	0.012^*^	1	0.070
4–5	95 (69.3)	42 (30.7)			1.011 (0.577–1.770)	0.970
≥6	85 (82.5)	18 (17.5)			2.063 (1.036–4.106)	0.039^*^
**Monthly household income**						
<₦20,000	84 (71.8)	33 (28.2)	2.541	0.468		
₦20,000–49,999	98 (69.5)	43 (30.5)				
₦50,000–99,999	63 (77.8)	18 (22.2)				
₦100,000+	16 (64.0)	9 (36.0)				

Abbreviations: AOR, adjusted odds ratio; CI, confidence interval.

* Statistically significant.

Place of residence was also a significant determinant, with children residing in rural areas being more than three times as likely to be fully immunized compared with their urban counterparts (AOR = 3.23; 95% CI: 1.79–5.83; *p* < 0.001). Household size showed a modest association; children from households with six or more members had higher odds of full immunization than those from smaller households (AOR = 2.06; *p* = 0.039). These findings highlight education, residential context, and family structure as key drivers of age‐appropriate childhood immunization.

The results indicate that child age and place of delivery were significant predictors of full immunization for age. The proportion of fully immunized children increased with age: only 50.5% of children aged 0–5 months were fully immunized, compared with 86.9% of those aged 18–23 months (*χ* ^2^ = 45.107, *p* < 0.001). After adjusting for potential confounders, children aged 12–17 months and 18–23 months were significantly more likely to be fully immunized than those aged 0–5 months (AOR = 5.42, 95% CI: 2.49–11.80; AOR = 6.83, 95% CI: 3.53–13.21, respectively; *p* < 0.001), highlighting the effect of age progression on vaccine completion (Table 2).

**TABLE 2:** Child's factors associated with immunization status.

Variable	Fully immunized for age		*χ* ^2^	*p* value	AOR (95 CI)	*p* value
	Yes (*n* = 261) Frequency (%)	No (*n* = 103) Frequency (%)				
**Child's age**						
0–5 months	48 (50.5)	47 (49.5)	45.107	<0.001^*^	1	<0.001^*^
6–11 months	38 (59.4)	26 (40.6)			1.506 (0.776–2.923)	0.226
12–17 months	56 (82.4)	12 (17.6)			5.417 (2.487–11.80)	<0.001^*^
18–23 months	119 (86.9)	18 (13.1)			6.828 (3.531–13.21)	<0.001^*^
**Sex of child**						
Male	151 (73.3)	55 (26.7)	0.597	0.440		
Female	110 (69.6)	48 (30.4)				
**Birth order**						
1st	76 (65.5)	40 (34.5)	6.867	0.076		
2nd–3rd	124 (71.7)	49 (28.3)				
4th–5th	49 (84.5)	9 (15.5)				
6th+	12 (70.6)	5 (29.4)				
**Place of delivery**						
Health facility	232 (76.3)	72 (23.7)	19.338	<0.001^*^	3.956 (2.107–7.427)	<0.001^*^
Home	29 (48.3)	31 (51.7)			1	

Abbreviations: AOR, adjusted odds ratio; CI, confidence interval.

* Statistically significant.

Place of delivery was also strongly associated with immunization status. Children delivered in health facilities had nearly four times higher odds of being fully immunized compared with those delivered at home (AOR = 3.96; 95% CI: 2.11–7.43; *p* < 0.001), emphasizing the critical role of facility‐based delivery in facilitating timely vaccination. Sex of the child and birth order were not significantly associated with full immunization.

The analysis of caregiver knowledge and perceptions revealed that several factors were significantly associated with children being fully immunized. Although almost all caregivers (99.6%) knew what immunization is, knowledge of the purpose of immunization showed a significant bivariate association (*χ* ^2^ = 5.094, *p* = 0.024), although this was not retained in multivariate analysis (AOR = 1.09; *p* = 0.921) (Table 3).

**TABLE 3:** Caregiver knowledge and perceptions on immunization factors associated with child's immunization status.

Variable	Fully immunized for age		*χ* ^2^	*p* value	AOR (95% CI)	*p* value
	Yes (*n* = 261) Frequency (%)	No (*n* = 103) Frequency (%)				
**Know what immunization is**						
Yes	260 (71.8)	102	0.467	1.000		
No	1 (50.0)	(28.2) 1 (50.0)				
**Purpose of immunization**						
To prevent diseases	257 (72.6)	97 (27.4)	5.094	0.024^*^	1.085 (0.219–5.369)	0.921
Others	4 (40.0)	6 (60.0)			1	
**Source of information about immunization**						
Health worker	219 (79.3)	57 (20.7)	38.053	<0.001^*^	1	<0.001^*^
Media	13 (68.4)	6 (31.6)			0.572 (0.206–1.586)	0.283
Family/Friends	21 (42.9)	28 (57.1)			0.214 (0.110–0.415)	<0.001^*^
Religious leader	8 (40.0)	12 (60.0)			0.186 (0.071–0.491)	<0.001^*^
**Age a child start immunization**						
At birth	260 (72.4)	99 (27.6)	6.679	0.010^*^	6.051 (0.509–71.877)	0.154
Others	1 (20.0)	4 (80.0)			1	
**Number of childhood immunizations before age 2**						
1–3	49 (83.1)	10 (16.9)	4.484	0.106		
4–6	111 (69.8)	48 (30.2)				
≥7	101 (69.2)	45 (30.8)				
**Vaccines safety**						
Yes	253 (72.7)	95 (27.3)	3.885	0.049^*^	1.145 (0.332–3.951)	0.830
No/Not sure	8 (50.0)	8 (50.0)			1	
**Believe vaccines are effective in preventing childhood diseases**						
Yes	249 (72.0)	97 (28.0)	0.237	0.627		
No/Not sure	12 (66.7)	6 (33.3)				
**Ever been discouraged from immunizing child**						
Yes	15 (53.6)	13 (46.4)	4.915	0.027^*^	0.763 (0.307–1.898)	0.561
No	246 (73.2)	90 (26.8)			1	

Abbreviations: AOR, adjusted odds ratio; CI, confidence interval.

* Statistically significant.

Source of information about immunization was strongly associated with full immunization. Children whose caregivers received information from health workers had the highest coverage (79.3%), whereas those informed by family/friends or religious leaders had substantially lower coverage. In multivariate analysis, information from media, family/friends, or religious leaders was associated with significantly lower odds of full immunization compared with health worker sources (AORs: 0.21–0.19; *p* < 0.001).

Other factors with significant bivariate associations included starting immunization at birth (*χ* ^2^ = 6.679; *p* = 0.010), perceived vaccine safety (*χ* ^2^ = 3.885; *p* = 0.049), and having ever been discouraged from immunizing (*χ* ^2^ = 4.915; *p* = 0.027), although these did not remain significant after adjustment. These findings highlight the critical role of health‐worker‐led education and early initiation of immunization in improving coverage.

The analysis of access to health services and barriers indicates that time taken to reach the immunization site, availability of regular community immunization sessions, and history of missed immunizations were significantly associated with full immunization for age (Table 4). Children whose caregivers required less than 30 min to reach the immunization site had higher odds of being fully immunized compared with those who took over an hour (AOR = 5.84; 95% CI: 1.33–25.55; *p* = 0.019), highlighting the impact of geographic accessibility. Similarly, the presence of regular immunization sessions within the community significantly increased the likelihood of full immunization (AOR = 2.76; 95% CI: 1.01–7.58; *p* = 0.049), emphasizing the importance of consistent service delivery close to households.

**TABLE 4:** Access to health services and barriers associated with child's immunization status.

Variable	Fully immunized for age		*χ* ^2^	*p* value	AOR (95% CI)	*p* value
	Yes (*n* = 261) Frequency (%)	No (*n* = 103) Frequency (%)				
**Distance to the immunization center**						
<1 km	3 (50.0)	3 (50.0)	4.779	0.092		
1–5 km	236 (70.9)	97 (29.1)				
>5 km	22 (88.0)	3 (12.0)				
**Usual means of transport**						
Walking	191 (72.6)	72 (27.4)	0.692	0.707		
Motorcycle	66 (68.8)	30 (31.3)				
Vehicle	4 (80.0)	1 (20.0)				
**Time taken to reach the immunization site**						
<30 min	216 (75.0)	72 (25.0)	10.758	0.005^*^	5.835 (1.332–25.554)	0.019^*^
30–59 min	42 (62.7)	25 (37.3)			3.451 (0.736–16.174)	0.116
1 h+	3 (33.3)	6 (66.7)			1	0.021^*^
**Have to pay for transport to the immunization site**						
Yes	93 (67.4)	45 (32.6)	2.037	0.154		
No	168 (74.3)	58 (25.7)				
**Health facility ever run out of vaccines**						
Yes	87 (72.5)	33 (27.5)	0.056	0.813		
No	174 (71.3)	70 (28.7)				
**Waiting times discouraging**						
Yes	100 (74.1)	35 (25.9)	0.594	0.441		
No	161 (70.3)	68 (29.7)				
**Health workers respectful**						
Yes	257 (71.6)	102 (28.4)	0.172	0.678		
No	4 (80.0)	1 (20.0)				
**Regular immunization sessions in community**						
Yes	253 (73.8)	90 (26.2)	12.406	<0.001^*^	2.763 (1.007–7.584)	0.049^*^
No	8 (38.1)	13 (61.9)			1	
**Ever missed immunization**						
Yes	18 (34.0)	35 (66.0)	43.548	<0.001^*^	0.165 (0.086–0.316)	<0.001^*^
No	243 (78.1)	68 (21.9)			1	

Abbreviations: AOR, adjusted odds ratio; CI, confidence interval.

* Statistically significant.

Conversely, children who had ever missed an immunization were significantly less likely to be fully immunized (AOR = 0.17; 95% CI: 0.09–0.32; *p* < 0.001), underscoring the cumulative effect of missed doses on overall coverage. Other factors, such as distance, transport mode, payment for transport, vaccine stock‐outs, waiting times, and health worker attitudes, were not significantly associated with full immunization in adjusted analyses. These findings highlight that proximity, regular service availability, and adherence to immunization schedules are critical determinants of complete childhood immunization.

Finally, Table 5 shows the analysis of caregiver attitudes and practices demonstrated that positive behaviors and supportive social influences were strongly associated with full immunization for age. Caregivers who reported that they would return for catch‐up immunization if a dose was missed had markedly higher odds of having fully immunized children (AOR = 8.74; 95% CI: 2.26–33.73; *p* = 0.002), highlighting the importance of adherence to vaccination schedules. Similarly, caregivers who would recommend immunization to other mothers in the community were significantly more likely to have fully immunized children (AOR = 3.22; 95% CI: 1.02–10.17; *p* = 0.046), indicating the influence of proactive health promotion behaviors.

**TABLE 5:** Attitudes and practices factors associated with child's immunization status.

Variable	Fully immunized for age		*χ* ^2^	*p* value	AOR (95% CI)	*p* value
	Yes (*n* = 261) Frequency (%)	No (*n* = 103) Frequency (%)				
If child missed a dose, would return for catch‐up immunization						
Yes No/Not sure	258 (74.6) 3 (16.7)	88 (25.4) 15 (83.3)	28.270	<0.001^*^	8.735 (2.262–33.730) 1	0.002^*^
**Would recommend immunization to other mothers in the community**						
Yes No/Not sure	255 (74.1) 6 (30.0)	89 (25.9) 14 (70.0)	18.140	<0.001^*^	3.224 (1.022–10.173) 1	0.046^*^
**Believe traditional remedies can replace vaccines**						
Yes No	48 (66.7) 213 (72.9)	24 (33.3) 79 (27.1)	1.122	0.289		
**Person who influences decision the most regarding immunization**						
Self Spouse Mother/Mother‐in‐law Religious leader Community leader	173 (78.3) 47 (70.1) 26 (57.8) 10 (55.6) 5 (38.5)	48 (21.7) 20 (29.9) 19 (42.2) 8 (44.4) 8 (61.5)	18.486	<0.001^*^	1 0.693 (0.364–1.319) 0.419 (0.206–0.852) 0.363 (0.131–1.011) 0.210 (0.062–0.714)	0.012^*^ 0.264 0.016^*^ 0.052 0.012^*^

Abbreviations: AOR, adjusted odds ratio; CI, confidence interval.

* Statistically significant.

Decision‐making dynamics also played a role: Children whose mothers’‐in‐law were the primary influencers on immunization decisions had lower odds of full immunization (AOR = 0.42; 95% CI: 0.21–0.85; *p* = 0.016), and influence from community leaders was similarly associated with reduced odds (AOR = 0.21; 95% CI: 0.06–0.71; *p* = 0.012), suggesting that reliance on non‐parental authority figures may impede timely vaccination. Belief in traditional remedies did not significantly affect immunization status. Overall, these findings underscore the critical role of caregiver engagement, health‐seeking attitudes, and maternal autonomy in promoting complete childhood immunization.

## 4 Discussion

This study provides comprehensive evidence on the multifactorial determinants of age‐appropriate childhood immunization, integrating caregiver sociodemographic characteristics, child‐related factors, knowledge and perceptions, access to health services, and attitudes and practices. In general, nearly three‐quarters of children were fully immunized for age, a coverage level that, while encouraging, still falls short of global and national targets required to achieve herd immunity against vaccine‐preventable diseases [26, 27, 28]. The findings underscore that immunization uptake is shaped not by a single factor but by intersecting individual, household, community, and health system influences.

Caregiver education emerged as one of the strongest and most consistent predictors of full immunization for age. Children whose caregivers had primary, secondary, or tertiary education were between three and five times more likely to be fully immunized compared with those whose caregivers had no formal education. This gradient effect suggests a dose–response relationship between educational attainment and immunization behavior, reinforcing evidence from multiple low‐ and middle‐income country settings [7]. Education likely enhances health literacy, improves comprehension of immunization schedules, and increases caregivers’ confidence in interacting with health services [7, 8, 9, 10]. Importantly, the magnitude of association observed in this study indicates that even basic education confers substantial benefits for child health, highlighting the broader intersectoral value of investments in female and caregiver education [17, 29, 30].

An unexpected but notable finding was the higher likelihood of full immunization among children residing in rural areas compared with urban residents. This contrasts with much of the existing literature, where urban residence is often associated with better immunization outcomes due to proximity to health facilities [31, 32, 33, 34]. However, this finding may reflect the effectiveness of targeted rural outreach services, community‐based immunization sessions, and stronger social cohesion in rural settings, which can facilitate information sharing and collective health‐seeking behaviors. Conversely, urban settings, particularly informal settlements, may face challenges such as population mobility, competing livelihood demands, and weaker continuity of care. This result emphasizes the need to disaggregate “urban” contexts and address urban inequities rather than assuming universal advantage.

Household size was also independently associated with immunization status, with children from households of six or more members being more likely to be fully immunized. Larger households may benefit from shared caregiving responsibilities, prior experience with immunization among older siblings, and accumulated knowledge of health services [35, 36, 37, 38]. This finding contrasts with studies reporting reduced immunization uptake in larger households due to resource constraints, suggesting that contextual factors, such as social support networks, may modify the effect of household size.

Child‐related factors showed strong associations with immunization status. Increasing child age was a powerful predictor of full immunization, with children aged 12–23 months having five‐ to sevenfold higher odds of being fully immunized compared with infants aged 0–5 months [39, 40]. This pattern reflects the cumulative nature of immunization schedules and highlights missed opportunities for timely vaccination in early infancy. Health facility delivery was another critical determinant, nearly quadrupling the likelihood of full immunization. Facility‐based delivery provides early contact with health workers, immediate initiation of vaccines, issuance of immunization cards, and counseling of caregivers, thereby establishing a continuum of care that extends into routine immunization.

Caregiver knowledge and information sources played a pivotal role in shaping immunization outcomes [40, 41, 42, 43, 44]. Although general awareness of immunization was high, the source of information significantly influenced uptake. Children whose caregivers relied on family, friends, or religious leaders for immunization information were substantially less likely to be fully immunized than those informed by health workers. This finding highlights the central role of trusted, technically competent health workers in countering misinformation and building vaccine confidence [41, 42]. It also underscores the potential risk posed by informal information channels if they are not aligned with evidence‐based messages.

Access‐related factors further illuminated structural barriers to immunization. Shorter travel time to immunization sites and the availability of regular community immunization sessions significantly increased the likelihood of full immunization. These findings align with health systems research emphasizing convenience, proximity, and service reliability as key drivers of service utilization [26]. Notably, children who had ever missed an immunization were dramatically less likely to be fully immunized, illustrating how early disruptions in vaccination schedules can have lasting consequences. This underscores the importance of reminder systems, defaulter tracking, and flexible catch‐up strategies.

Attitudes and practices were among the most powerful predictors observed in this study. Caregivers who intended to seek catch‐up immunization after missed doses had almost ninefold higher odds of having fully immunized children, whereas willingness to recommend immunization to others also significantly increased uptake. These findings suggest that positive normative beliefs and proactive health‐seeking intentions are critical levers for improving immunization coverage [45, 46, 47]. Conversely, decision‐making dominated by extended family members or community leaders was associated with reduced likelihood of full immunization, reflecting the complex influence of social hierarchies and cultural norms on health behaviors [18].

Taken together, these findings have important implications for policy and practice. Interventions to improve childhood immunization should adopt a multidimensional approach that strengthens caregiver education and health literacy, reinforces health‐worker‐led communication, promotes facility‐based deliveries, and expands regular, community‐based immunization services. Engaging influential community figures with accurate, context‐sensitive information may help transform them from barriers into facilitators of immunization. Addressing early missed vaccinations through robust follow‐up and catch‐up strategies is particularly critical for sustaining full immunization coverage.

### 4.1 Strengths and Limitations of the Study

A key strength of this study lies in its comprehensive analytical approach, which examined childhood immunization determinants across multiple domains, including caregiver sociodemographic characteristics, child‐related factors, knowledge and perceptions, access to health services, and caregiver attitudes and practices. This multidimensional framework provides a nuanced understanding of the complex pathways influencing age‐appropriate immunization and enhances the relevance of the findings for policy and programmatic decision‐making. The use of multivariable logistic regression allowed for adjustment of potential confounders, strengthening the internal validity of the identified associations. In addition, verification of immunization status using vaccination cards, where available, improved data accuracy and reduced misclassification bias.

Despite these strengths, several limitations should be acknowledged. The cross‐sectional design precludes causal inference, as exposure and outcome were assessed simultaneously. Immunization status for children without vaccination cards relied partly on caregiver recall, which may be subject to recall and social desirability biases. The study was conducted in a specific geographic context, which may limit the generalizability of the findings to other settings with different health system structures or sociocultural dynamics. Furthermore, some potentially important factors, such as health worker workload and facility‐level service quality indicators, were not directly measured. These limitations should be considered when interpreting the findings, although they do not detract from the study's overall contribution to understanding immunization uptake.

## 5 Conclusion

This study demonstrates that full, age‐appropriate childhood immunization is shaped by a complex interplay of caregiver characteristics, child‐related factors, health system accessibility, and social and behavioral influences. Caregiver education emerged as a particularly strong determinant, underscoring the critical role of health literacy in promoting timely and complete vaccination. Child age and place of delivery further highlighted the importance of early and continuous engagement with health services, especially through facility‐based deliveries that facilitate linkage to routine immunization. Access‐related factors, including travel time to immunization sites and the availability of regular community immunization sessions, significantly influenced uptake, emphasizing the need for convenient and reliable service delivery. Attitudes and practices, such as intention for catch‐up immunization, peer recommendation, and decision‐making autonomy, were among the most powerful predictors of full immunization, revealing the central role of caregiver motivation and social influence. To achieve and sustain high immunization coverage, interventions must go beyond vaccine availability to address educational, behavioral, and structural barriers simultaneously. Strengthening health‐worker‐led communication, promoting community‐based immunization strategies, and supporting caregivers to complete missed doses are essential for closing persistent coverage gaps. An integrated, people‐centered approach is, therefore, critical to advancing equitable childhood immunization and improving child survival outcomes.

## Author Contributions

Aruwa Simon Ayigu, Stephen Olaide Aremu, and Adamu Ishaku Akyala conceptualized this study. Aruwa Simon Ayigu, Stephen Olaide Aremu, and Adamu Ishaku Akyala conducted a literature search to put together relevant studies. All authors wrote the initial draft, which Stephen Olaide Aremu and Adamu Ishaku Akyala edited. Stephen Olaide Aremu is the primary corresponding author, and Adamu Ishaku Akyala and Stephen Olaide Aremu are responsible for the work's credibility.

## Funding

The authors have nothing to report.

## Ethics Statement

Ethical approval was obtained from the Health Research Ethics Committee of the Nasarawa State Ministry of Health. Participation was voluntary, anonymity was ensured. The study adhered to the ethical principles outlined in the Declaration of Helsinki. Permission was also secured from relevant community and health authorities prior to data collection.

## Consent

Consent for participation was collected from all study participants prior to their involvement in the research. Participants were informed of the study's purpose, procedures, and potential risks. They were assured that their participation was voluntary and that their personal information would remain confidential. Written consent was obtained before data collection began.

## Conflicts of Interest

The authors declare no conflicts of interest.

## Data Availability

The datasets generated and analyzed during the current study are not publicly available due to privacy considerations of the participants but are available from the corresponding author upon reasonable request.
